# Virological outcome and patterns of HIV-1 drug resistance in patients with 36 months’ antiretroviral therapy experience in Cameroon

**DOI:** 10.7448/IAS.16.1.18004

**Published:** 2013-01-31

**Authors:** Avelin F Aghokeng, Charles Kouanfack, Sabrina Eymard-Duvernay, Christelle Butel, Ginette E Edoul, Christian Laurent, Sinata Koulla-Shiro, Eric Delaporte, Eitel Mpoudi-Ngole, Martine Peeters

**Affiliations:** 1 Virology Laboratory CREMER/IMPM/IRD, Yaoundé, Cameroon; 2 UMI 233 TransVIHMI, Institut de Recherche pour le Développement (IRD), University Montpellier 1, Montpellier, France; 3 Central Hospital, Yaoundé, Cameroon

**Keywords:** HIV-1, treatment outcome, virological monitoring, drug resistance, resource-limited country, Cameroon

## Abstract

**Introduction:**

The current expansion of antiretroviral treatment (ART) in the developing world without routine virological monitoring still raises concerns on the outcome of the strategy in terms of virological success and drug resistance burden. We assessed the virological outcome and drug resistance mutations in patients with 36 months’ ART experience, and monitored according to the WHO public health approach in Cameroon.

**Methods:**

We consecutively recruited between 2008 and 2009 patients attending a national reference clinic in Yaoundé – Cameroon, for their routine medical visits at month 36±2. Observance data and treatment histories were extracted from medical records. Blood samples were collected for viral load (VL) testing and genotyping of drug resistance when HIV-1 RNA≥1000 copies/ml.

**Results:**

Overall, 376 HIV-1 infected adults were recruited during the study period. All, but four who received PMTCT, were ART-naïve at treatment initiation, and 371/376 (98.7%) started on a first-line regimen that included 3TC +d4T/AZT+NVP/EFV. Sixty-six (17.6%) patients experienced virological failure (VL≥1000 copies/ml) and 53 carried a resistant virus, thus representing 81.5% (53/65) of the patients who failed. Forty-two out of 53 were resistant to nucleoside and non-nucleoside reverse-transcriptase inhibitors (NRTIs+NNRTIs), one to protease inhibitors (PI) and NNRTIs, two to NRTIs only and eight to NNRTIs only. Among patients with NRTI resistance, 18/44 (40.9%) carried Thymidine Analog Mutations (TAMs), and 13/44 (29.5%) accumulated at least three NRTI resistance mutations. Observed NNRTI resistance mutations affected drugs of the regimen, essentially nevirapine and efavirenz, but several patients (10/51, 19.6%) accumulated mutations that may have compromised etravirine use.

**Conclusions:**

We observed a moderate level of virological failure after 36 months of treatment, but a high proportion of patients who failed developed drug resistance. Although we found that for the majority of patients, second-line regimens recommended in Cameroon would be still effective, accumulated resistance mutations are of concern and may compromise future treatment strategies, stressing the need for virological monitoring in resource-limited settings.

## Introduction

Antiretroviral therapy (ART) has significantly reduced morbidity and mortality in human immunodeficiency virus type 1 (HIV-1)-positive patients in both industrialized and resource-poor countries. Because ART can fail as a result of toxicity, pretreatment HIV-1 drug resistance, insufficient patient adherence or incomplete suppression of viral replication leading to the emergence of drug-resistant viruses, adequate clinical and biological management can significantly improve treatment outcome and can prevent rapid failure [1,2]. Current World Health Organisation (WHO) recommendations favour the use of viral load monitoring [3], but its practical feasibility is still challenging in the context of resource-poor countries, essentially because of the high cost. Drug resistance evaluation can provide helpful information for treatment switch by guiding the selection of appropriate ARV regimens when a treatment failure is diagnosed, but the technology and assays are still very expensive and hard to implement locally due to inadequate infrastructures and lack of specialized personnel.

Despite these limitations in ART access and monitoring, recent studies assessing the outcome of ART in the developing world have shown significantly good results, with good virological success achieved after 12 and/or 24 months of ART, and even limited consequences of observed drug resistance mutations for second-line options [4,5]. In addition, few clinical trials, comparing both the clinical plus laboratory-based approach versus the public health monitoring approach alone, have not clearly identified significant differences in terms of viral suppression and the emergence of drug resistant strains, as well as deaths [6,7]. The main limitation of some of these studies is the short period of evaluation, and, therefore, little is known about the long-term consequences of this strategy in terms of the accumulation of drug resistance mutations and possible consequences for second- and/or third-line treatments.

Since the 2000s, ART access in Cameroon has been significantly improved through the implementation of the WHO simplify approach and the decentralization of ART services. The standard first-line therapy consists of two nucleoside reverse transcriptase inhibitors (NRTIs) and one non-NRTI (NNRTI), and until 2010 when WHO recommended the replacement of stavudine with tenofovir, reference first-line antiretrovirals (ARVs) in Cameroon included zidovudine or stavudine plus lamivudine as NRTIs and nevirapine or efavirenz as NNRTIs. First- and second-line treatments have been freely provided to eligible patients since 2007, and treatment initiation and monitoring has been guided by clinical and/or immunological data.

In this study, we evaluated the long-term virological outcome and implications for second-line regimens after 36 months’ ART in patients treated according to the WHO public health approach in Cameroon.

## Methods

### Study site and patients

From September 2008 to September 2009, we conducted a cross-sectional study among ARV-treated patients attending a reference treatment unit, the “Hôpital de Jour” of the Yaoundé Central Hospital. In this unit, patients received ART as per national recommendations, and the decision to treat or switch is mostly guided by clinical and/or immunological assessments. We consecutively enrolled 376 HIV-positive adults who attended the clinic for their follow-up visit after 36 months’ ART (±two months). Patients who agreed to participate were invited to sign an informed consent, and we administrated a standardized questionnaire to collect patients’ demographic, epidemiological and clinical data. We collected information on the last CD4 count test performed and the number of viral load (VL) done since ART start. The Cameroonian National Ethics Committee approved the study, and efforts were made to guarantee patients confidentiality throughout the study.

### Sample processing, viral load and genotyping testing

We collected from each patient 10 mL of whole blood from which plasma specimens were recovered after centrifugation and stored at −80°C. Because of the circulation of almost all identified HIV-1 groups in Cameroon and documentation of few HIV-2 strains, we performed an ELISA-based serotyping assay to differentiate between HIV-1 group M, N and O infections, as well as between HIV-1 and 2 infections [8]. We quantified HIV-1 RNA load from each patient using a second-generation real-time RT-PCR assay (Biocentric, Ze Val d'Aran, Bandol) with a lower detection limit of 300 copies/ml [9]. A virologic failure was considered for all patients with HIV-1 RNA≥1000 copies/ml, and for these patients, a drug resistance testing was performed using a previously described home-brew assay optimized for HIV-1 non-B strains. Briefly, viral RNA was extracted from the plasma using the QIAamp Viral RNA kit (Qiagen, Courtaboeuf, France), and amplified fragments covering the viral protease (amino acids 1–99) and reverse transcriptase (amino acids 1–310) were generated with outer G25REV-IN3 and inner AV150-polM4 primers. The assay detected mutants that comprise at least 20% of the virus population [10]. Amino acid sequences were analyzed to identify relevant drug resistance mutations (DRMs), using the Agence Nationale de Recherche sur le Sida et les Hépatites (ANRS) interpretation algorithm, version May 2011 (http://www.hivfrenchresistance.org/).

### Nucleotide sequence accession numbers

The newly reported protease and reverse transcriptase sequences are available in GenBank under the following accession numbers: JX982979 to JX983043.

## Results

### Patients’ demographic and clinical characteristics

Within the study period, we recruited 376 HIV-1 infected adults, who met the criteria of having 36±2 months’ ART experience and who were attending the clinic for their routine monitoring visit. Two-thirds of these patients (274/376, 72.9%) were women, and the median age (interquartile range) was 40 (33–47) years. Almost all patients performed a CD4-T cells count within the last six months before recruitment, and the median CD4-T cells count (interquartile range) was relatively high: 373 (221–502) cells/µl. WHO clinical stages varied from stage 1 to stage 4, the majority of patients being at stage 3, 142 patients (37.8%), followed by stage 2, 4 and 1 at 90 (23.9%), 79 (21.0%) and 4 patients (1.1%), respectively (Table 1). We found no significant difference by sex; median age and CD4-T cells count were 43 (35–51) years and 355 (194–494) cells/µl, respectively, for males and 39 (32–46) years and 379 (232–509) cells/µl, respectively, for women. Only a few patients, 15 patients out of 376 (4%), performed a VL testing during their ART.

**Table 1:** Demographic and clinical characteristics of the study population

Characteristics	Month 36±2		

Men	Women	Total
Sex	102 (27.1%)	274 (72.9%)	376
Median age (IQR)	43 (35–51)	39 (32–46)	40 (33–47)
Median CD4, cells/µl	355 (194–494)	379 (232–509)	373 (221–502)
Viral load performed during ART	4 (3.9%)	11 (4.0%)	15 (4.0%)
WHO stages			
1	0 (0.0%)	4 (1.7%)	4 (1.1%)
2	25 (29.4%)	65 (28.3%)	90 (28.6%)
3	35 (41.2%)	107 (46.5%)	142 (45.1%)
4	25 (29.4%)	54 (23.5%)	79 (25.1%)
Not available	17 (16.6%)	44 (16.1%)	61 (16.2%)
ARV exposure prior to treatment initiation			
Previous ART	0	0	0
PMTCT	–	4 (1.1%)	4 (1.1%)
First-line ART			
3TC+D4T+NVP	41 (40.2%)	118 (43.1%)	159 (42.3%)
3TC+AZT+EFV	39 (38.2%)	68 (24.8%)	107 (28.5%)
3TC+D4T+EFV	20 (19.6%)	72 (26.3%)	92 (24.5%)
3TC+AZT+NVP	0 (0.0%)	13 (4.7%)	13 (3.5%)
3TC+DDI+EFV	2 (2.0%)	1 (0.4%)	3 (0.8%)
IDV+3TC+AZT	0 (0.0%)	2 (0.7%)	2 (0.5%)
Treatment switches			
Once	35 (34.3%)	134 (48.9%)	169 (44.9%)
Twice	10 (9.8%)	43 (15.7%)	53 (14.1%)
Three times	1 (1.0%)	14 (5.1%)	15 (4.0%)
Four times	0 (0.0%)	2 (0.7%)	2 (0.5%)
Treatment interruptions	11 (10.8%)	35 (12.8%)	46 (12.2%)
Main reasons for switch and/or interruption			
Side effects	13	27	40
Stock-outs	7	32	39
Pregnancy	–	12	12
Voluntary interruption	3	9	12
Lack of information	0	8	8

IQR, interquartile range; ART, antiretroviral therapy; WHO, World Health Organization; PMTCT, prevention of mother to child transmission; 3TC, lamivudine; d4T, stavudine; AZT, zidovudine; NVP, nevirapine; EFV, efavirenz; ddI, didanosine; IDV, indinavir.

### Antiretroviral treatment and adherence data

None of the recruited patients reported previous exposure to ARVs before the initiation of ART, except for four women who received ARVs as prevention of mother to child transmission of HIV (PMTCT). PMTCT regimens included nevirapine only (one patient), zidovudine plus nevirapine (two patients) and stavudine plus lamivudine plus nevirapine (one patient). Three hundred and seventy one (98.7%) patients received lamivudine plus stavudine/zidovudine plus nevirapine/efavirenz, and only five (1.3%) patients received other regimens, including lamivudine plus didanosine plus efavirenz and indinavir plus lamivudine plus zidovudine (Table 1). About half of the patients (169, 44.9%) experienced at least one treatment change, while two, three and four changes were reported for 53 (14.1%), 15 (4.0%), and 2 (0.5%) patients, respectively. These changes did not correspond to treatment line modification, but only substitution of first-line ARVs with other first-line molecules. Only 10 patients switched to second-line therapy because of diagnosed failure, and the second-line regimen contained 2NRTIs and one boosted protease inhibitor (PI/r), generally lamivudine/tenofovir plus abacavir/zidovudine plus indinavir/lopinavir boosted with ritonavir. The main reasons for ARV substitutions were adverse effects, stock-outs, pregnancy, voluntary interruption, and lack of information. In addition, 46 (12.2%) patients experienced at least one treatment interruption since the start of ART.

### Virological failure and HIV-1 drug resistance

We measured HIV-1 RNA level for all patients (*n*=376) to evaluate virologic outcome after 36 months’ ART. All patients carried HIV-1 group M virus. Two hundred and ninety out of the recruited 376 (77.1%) patients had a VL level bellow the assay detection limit, 300 copies/ml, and the median HIV-1 RNA level for results>300 copies/ml was 4.79 (3.33–5.55) Log10 copies/ml (Table 2). Sixty-six (17.6%) had plasma HIV-1 RNA level≥1000 copies/ml and underwent drug resistance genotyping to investigate the presence of a resistant virus. We found an equal distribution according to gender: 17 (16.7%) were males and 49 (17.9%) were women. Of the four women who received PMTCT, only one failed with a VL of 4.9 Log10 copies/ml. Successful genotyping was achieved for 65 of the 66 samples tested, and for one sample (HIV-1 RNA level of 1045 copies/ml) all genotyping attempts failed. The identified viral strains included CRF02_AG (*n*=44), A (*n*=7), D (*n*=3), F2 (*n*=3), CRF11_cpx (*n*=3), C (*n*=1), G (*n*=1), H (*n*=1), CRF06_cpx (*n*=1) and other recombinant (*n*=1). Among the 65 patients with successful genotyping, 53 (81.5%) carried a virus with at least one major DRM. Up to 73.6% (39/53) were women, thus correlating with the gender ratio of our study population.

**Table 2:** HIV-1 drug resistance after 36 months of antiretroviral therapy in patients with viral load ≥ 1000 copies/ml

Variable	Month 36±2 (*n*=376)		

	Men	Women	Total
Viral load			
Viral load≥300 copies/mlα	25 (24.5%)	61 (22.3%)	86 (22.9%)
Viral load, median Log10 copies/mlβ	2.9 (2.5–5.0)	4.8 (3.5–5.5)	4.8 (3.3–5.6)
Viral load≥1000 copies/ml	17 (16.7%)	49 (17.9%)	66 (17.6%)
Genotypic HIV-1 drug resistance			
Successful genotyping of samples with viral load≥1000 copies/ml	17/17	48/49	65/66
Presence of ≥1 major drug resistance mutation	14/17 (82.4%)	39/48 (81.3%)	53/65 (81.5%)
Affected ARV regimens			
D4T only	0	1	1
ETR only	0	1	1
3TC/FTC only	0	1	1
NVP/EFV only	1	6	7
3TC/FTC+NVP/EFV	5	13	18
IDV+NFV+NVP/EFV	1	0	1
3TC/FTC+NVP/EFV+ETR	0	4	4
3TC/FTC+AZT+D4T+NVP	0	1	1
3TC/FTC+AZT+D4T+NVP/EFV	3	5	8
3TC/FTC+ABC+NVP/EFV+ETR	1	0	1
3TC/FTC+AZT+D4T+NVP/EFV+ETR	1	2	3
3TC/FTC+AZT+D4T+TDF+NVP/EFV	0	1	1
3TC/FTC+AZT+D4T+ABC+TDF+NVP/EFV	1	4	5
3TC/FTC, AZT, d4T, ABC, TDF+NVP/EFV+ETR	1	0	1
Mutations associated with PI resistance			
L90M	1	0	1
Mutations associated with NRTI resistance			
V75M	0	1	1
M184V	5	18	23
M184V, L74V	1	0	1
M184V, T215FIY	3	3	6
M184V, M41L, T215Y	1	0	1
M184V, K70R, T215F	0	1	1
M184V, M41L, V75I, T215F	1	0	1
M184V, K70R, T215F, K219Q	0	1	1
M184V, D67DN, K70R, T215F	0	1	1
M184V, M41L, L210W, T215FY	1	3	4
M184V, M41L, D67N, L210W, T215Y	1	0	1
M184V, D67N, K70R, T215FY, K219EQ	0	2	2
M184V, M41L, D67DN, V75M, L210W, T215Y	0	1	1
Mutations associated with NNRTI resistance			
K103N	5	13	18
Y181C	1	4	5
G190AS	0	3	3
E138A	0	1	1
Y188L	1	0	1
K103N, P225HY	1	5	6
Y181C, H221Y	1	4	5
V106IM, Y188HL	1	1	2
K103N, M230L	0	1	1
K101H, G190A	0	1	1
G190S, H221Y	1	0	1
G190A, M230L	0	1	1
V106M, F227L	0	1	1
Y181C, G190A	0	1	1
K103N, Y181C, H221Y	1	0	1
K103N, H221Y, M230L	1	0	1
V106A, Y181C, H221Y	1	0	1

α 300 copies/ml represents the assay lower detection limit.

β The median viral load level was calculated for results>300 copies/ml.

3TC, lamivudine; FTC, emtricitabine; d4T, stavudine; AZT, zidovudine; ABC, abacavir; TDF, tenofovir; NVP, nevirapine; EFV, efavirenz; ddI, didanosine; IDV, indinavir; ETR, etravirine.

Overall, 42 patients were resistant to both NRTIs and NNRTIs, 12 (28.6%) were male and 30 (71.4%) were women. One patient was resistant to PIs and NNRTIs, two women to NRTIs only and eight (one male and seven women) to NNRTIs only. Only one patient had a PI resistance mutation, L90M, and although this patient experienced several treatment substitutions, he received no PI. Almost all NRTI resistant patients carried the M184V mutation (43 out of 44), and 18 (40.9%) carried at least one Thymidine Analog Mutation (TAM); 13 (29.5%) accumulated at least three NRTI DRM, including several TAMs and, therefore, developed resistance to drugs of their regimen as lamivudine/emtricitabine or stavudine, but this accumulation also compromised other drugs that were not included in ART regimens as tenofovir and/or abacavir (Table 3). Among the 53 patients with any DRM, 51 (96.2%) developed resistance to NNRTIs and the most prevalent mutations included K103N, Y181C, G190AS, H221Y and P225HY. Observed profiles included not only single or multiple DRMs that affected both nevirapine and efavirenz for the majority of patients but also other NNRTIs that were not included in prescribed regimens as etravirine. We found etravirine resistance for 10 patients out of the 51 with NNRTI resistance (19.6%), and for 7 of them, resistance was associated with the presence of 2 frequently observed NNRTI resistance mutations, Y181C and H221Y (Table 3). Among the 10 patients who switched to a second-line regimen, 2 showed virologic failure and both were recruited at least two months after the initiation of the second-line ART. One had a wild type virus with a VL of 6.6 Log10 copies/ml, indicating an undeclared treatment interruption, and the second developed resistance to one NRTI of his second-line regimen but showed no PI mutation.

**Table 3:** Mutation patterns associated with resistance to ARVs not included in ART regimens

Patients	Sequentially received ARV regimens	Mutation profiles	Affected first-line ARVs	Other compromised ARVs	Sex (M/F)α
NRTI-resistance profiles					
1	NVP/D4T/3TC	M184V, A62AV, L74LV	3TC/FTC	ABC	M
2	EFV/D4T/3TC, EFV/AZT/3TC	M184V, M41L, L210W, T215Y	3TC/FTC, AZT, d4T	ABC, TDF	F
3	EFV/D4T/3TC, EFV/AZT/3TC	M184V, M41L, L210W, T215Y	3TC/FTC, AZT, d4T	ABC, TDF	F
4	NVP/D4T/3TC, NVP/AZT/3TC, DDI/AZT/3TC	M184V, M41L, L210W, T215Y	3TC/FTC, AZT, d4T	ABC, TDF	F
5	EFV/D4T/3TC	M184V, M41L, L210W, T215F	3TC/FTC, AZT, d4T	ABC, TDF	M
6	NVP/D4T/3TC	M184V, D67DN, T69D, K70KR, T215F	3TC/FTC, AZT, d4T	TDF	F
7	EFV/AZT/3TC	M184V, M41L, D67DN, V75M, L210W, T215Y	3TC/FTC, AZT, d4T	ABC, TDF	F
NNRTI-resistance profiles					
1	EFV/D4T/3TC, EFV/AZT/3TC, NVP/AZT/3TC	E138A		ETR	F
2	NVP/AZT/3TC, AZT/3TC/IDV, NVP/D4T/3TC	Y181C, H221Y	NVP, EFV	ETR	F
3	NVP/D4T/3TC	Y181C, H221Y	NVP, EFV	ETR	F
4	NVP/D4T/3TC, NVP/AZT/3TC, TDF/3TC/ABC/LPVr	Y181C, H221Y	NVP, EFV	ETR	M
5	NVP/D4T/3TC, EFV/AZT/3TC	Y181C, H221Y	NVP, EFV	ETR	F
6	EFV/D4T/3TC, NVP/D4T/3TC	Y181C, H221HY	NVP, EFV	ETR	F
7	EFV/D4T/3TC	K103N, Y181C, H221Y	NVP, EFV	ETR	M
8	NVP/D4T/3TC	V106A, Y181C, H221HY	NVP, EFV	ETR	F
9	NVP/AZT/3TC	V90IV, K101KR, V179I, Y181C*	NVP, EFV	ETR	F
10	NVP/D4T/3TC	A98AG, V179IT, G190S, H221HY*	NVP, EFV	ETR	M

3TC, lamivudine; FTC, emtricitabine; d4T, stavudine; AZT, zidovudine; ABC, abacavir; TDF, tenofovir; NVP, nevirapine; EFV, efavirenz; ddI, didanosine; IDV, indinavir; ETR, etravirine.

α Male or female.

* Polymorphic mutations (V90I, A98G, K101R and V179IT) contributed to resistance or possible resistance to etravirine.

## Discussion

We reported in this study the virological outcome and HIV drug resistance among patients followed-up in a national HIV treatment centre in Yaoundé – Cameroon, and who stayed under ART for 36±2 months. In the same clinic, we conducted a cross-sectional study in 2006–2007 to assess ART outcome and selection of drug-resistant viruses after 12 or 24 months of ART and found virological failures (VL≥1000 copies/ml) of 16.4% (41/249) and 22.5% (40/178), respectively, at months 12 and 24. We showed in that previous study that at month 24, up to 79% (30/38) of patients who failed, representing 16.9% of all patients recruited at month 24, carried at least one major DRM, while only 32.4% (11/34), 4.4% of all patients at month 12, developed drug resistance, thus illustrating significant adherence issues for these patients, since the majority of those failing treatment after 12 months had no resistant virus [5]. We did not recruit the same patients for the present study, and the results obtained are promising because we observed no increase of virological failure for patients at M36, as that could be anticipated if we consider observations from months 12 and 24. In fact, we found a failure rate of 17.6% (66/376) in patients with 36 months’ ART experience, which is lower than the result obtained for patients who had 24 months ART (22.5%) [5]. In addition, the proportion of patients with a resistant virus did not increase significantly, here we reported 81.5% and we previously found 79% for M24 patients. This improvement of ART outcome is either the result of a better management of patients by clinical staff who are gaining more experience over time, and/or a bias due to the cross-sectional design of the study which allows assess only to patients who are still being treated, and therefore unlikely to represent lost to follow up, ART stop and deaths. In addition, this result may indicate that failure due to adherence issues occurs early at treatment initiation and stabilizes over time among long-term treated patients, therefore advocating the need for early introduction of VL testing to identify non-adherent patients.

However, in this study we found that patients failing ART with a resistant virus significantly accumulated DRMs, especially NRTI mutations and TAMs, probably because they stayed longer under failing regimens, correlating with similar studies in Africa [11–13]. As already shown, this accumulation of NRTI/TAMs and also NNRTI mutations led to reduced sensitivity to drugs that were not part of the regimen as tenofovir, abacavir and etravirine [13,14], but did not significantly compromise currently recommended PI-based second-line regimens. In Cameroon, reference second-line regimens for adults consist of one boosted PI, routinely lopinavir or atazanavir boosted with ritonavir, plus two NRTIs among tenoforvir, lamivudine, zidovudine and emtricitabine, excluding when feasible, NRTIs already prescribed at first-line. In our study, only 7 patients out of the 53 who developed resistance, thus representing 1.9% (7/376) of our total study population, may be at risk of having a suboptimal second-line ART if they receive tenofovir and/or abacavir. However, up to 14% (53/376) of the patients will require a second-line treatment and subsequently third-line ARVs, in a context where access to these drug classes is still extremely challenging, especially for third-lines. In these conditions, there is a risk that patients failing second-lines will stay longer under a failing regimen and may develop additional DRMs that will be transmitted also. That represents a major public health threat for developing countries and thus advocates the need for additional efforts to improve first-line treatment outcome and prevent drug resistance. Although the study was not initially designed to compare gender response to ART treatment or evaluated ART failure according to gender, the analyses we performed did not show any significant difference between males and females. As observed in routine practice, women predominated in our study population, but the proportion of virological failure in both groups was very similar. Detailed assessment of drug resistance mutations showed no specific pattern that can characterise any of the two populations. In addition, for the four women who received ARVs as PMTCT intervention before their ART initiation, we found no specific results since only one failed after 36 months of treatment. However, the limited number of patients did not allow us to draw any conclusion.

We had one patient with a PI mutation, L90M, with an unclear origin since the patient did not receive any PI. Our analysis was limited by the lack of baseline data, including genotypic information before ART initiation, but threshold surveys conducted within the study period in Yaoundé revealed a low (<5%) level of transmitted PI-resistance mutations and low to moderate (5%–15%) rates of transmitted RTI-resistance mutations [15]. However, it is still likely that the observed L90M mutation resulted from transmitted resistance as we recently published in Cameroon [16], or from unreported exposure to PIs. Etravirine is currently not recommended in Cameroon as a second-line ARV, but could potentially serve as second- or third-line option, or could be used for patients who initiated ART before implementation of national programmes and who experienced several informal ARV regimens between the 1990s and 2000s. Our study and other reports from African settings [11,14] showed that accumulated NNRTI-resistance mutations might impair etravirine efficacy, thus compromising its use in NNRTI-experienced patients.

Contrary to several recent reports assessing failure to stavudine-containing first-line regimens in developing countries, we found no K65R mutation or Q151M complex known as compromising almost all NRTIs [13,17]. Several reasons have been suggested to explain the selection of K65R under tenofovir-free regimens, including delay in treatment switch, viral load level and subtype mediated pathway, but the main reasons are still uncertain [13,18–20]. However, some reports have suggested antagonism between K65R and TAMs, indicating that both pathways are unlikely to occur simultaneously. Indeed a large database analysis involving up to 66,000 genotypes found that K65R shows a strong negative association with specific TAMs including M41L, D67N, L210W, T215F/Y, and K219Q/E [21]. The high frequency of TAMs observed in our study could thus explain the fact that we found no K65R mutation, but other factors cannot be fully excluded.

## Conclusions

In conclusion, studies evaluating virological outcome after 36 months of ARV treatment in patients routinely managed in national ART programmes from developing countries are currently rare. Despite the absence of routine virological management, we did not find a dramatic level of failure. Also, we showed that second-line ART will work well for the majority of patients who developed resistance, and although illustrating the situation of a specific clinic and not the entire national programme, these results indicate that even under the public health approach, long-term virological success could be achieved for the majority of patients. Nonetheless, significant challenges still exist and should be rapidly addressed. Rapid identification of failure to prevent accumulation of resistance mutations is essential, and availability of simple and affordable viral load tools is important. In addition, programme management should be improved to reduce unnecessary drug substitutions due to drug stock-outs, voluntary interruptions and ignorance as we reported here.
